# Restless leg syndrome in multiple sclerosis: a case–control study

**DOI:** 10.3389/fneur.2023.1194212

**Published:** 2023-06-19

**Authors:** Salman Aljarallah, Nuha Alkhawajah, Omar Aldosari, Mohammed Alhuqbani, Faisal Alqifari, Bassam Alkhuwaitir, Abdullah Aldawood, Omar Alshenawy, Ahmed S. BaHammam

**Affiliations:** ^1^College of Medicine, King Saud University, Riyadh, Saudi Arabia; ^2^Department of Medicine, King Saud University, Riyadh, Saudi Arabia; ^3^The University Sleep Disorders Center, College of Medicine, King Saud University, Riyadh, Saudi Arabia; ^4^National Plan for Science and Technology, College of Medicine, King Saud University, Riyadh, Saudi Arabia

**Keywords:** restless leg syndrome (RLS) in multiple sclerosis, multiple sclerosis, prevalence, sleep disturbance, fatigue

## Abstract

**Objectives:**

This study assessed the prevalence of restless leg syndrome (RLS) among patients with multiple sclerosis (pwMS) and the association between RLS and MS disease duration, sleep disturbance, and daytime fatigue.

**Methods:**

In this cross-sectional study, we interviewed 123 patients via phone calls using preset questionnaires containing the International Restless Legs Syndrome Study Group (IRLSSG) diagnostic criteria, Pittsburgh Sleep Quality Index (PSQI), and Fatigue Severity Scale (FSS) diagnostic criteria validated in both Arabic and English. The prevalence of RLS in MS was compared to a group of healthy controls.

**Results:**

The prevalence of RLS in pwMS, defined by meeting all four requirements included in the IRLSSG diagnostic criteria, was 30.3% compared to 8.3% in the control group. About 27.3% had mild RLS, 36.4% presented with moderate, and the remaining had severe or very severe symptoms. Patients with MS who experience RLS had a 2.8 times higher risk of fatigue compared to pwMS without RLS. pwMS with RLS had worse sleep quality, with a mean difference of 0.64 in the global PSQI score. Sleep disturbance and latency had the most significant impact on sleep quality.

**Conclusion:**

The prevalence of RLS among MS patients was significantly higher compared to the control group. We recommend educating neurologists and general physicians to increase their awareness of the increasing prevalence of RLS and its association with fatigue and sleep disturbance in patients with MS.

## 1. Introduction

Multiple Sclerosis (MS) is an immune-mediated disease of the central nervous system that affects the myelin covering of the axons, which leads to disability (1). MS is gaining more attention worldwide because of its increasing prevalence across the globe (2). Over the past century, developments in the field have resulted in a marked improvement in prognosis and long-term outcomes. Nevertheless, certain aspects of the disease’s spectrum remain poorly understood (3, 4). Patients with MS can go through a multitude of symptoms that impact their day-to-day functioning and quality of life. While disability in MS has been traditionally attributed to walking impairment, imbalance, or visual difficulties, other less visible symptoms might equally impact the quality of life. For example, disturbances in sleep and fatigue are well-recognized by patients as prominent symptoms and have been shown to negatively impact patients’ quality of life (5). These symptoms are not uncommon among patients with MS (pwMS). Some studies have reported the prevalence of fatigue among pwMS to be more than 70% (6, 7). Also, disturbances in the quality of sleep have been reported to affect up to 25–50% of pwMS (8, 9). The etiology of sleep disturbances in patients with MS is not well identified and is likely to be multifactorial, and it includes symptoms such as pain, sensory disturbances, nocturia, and anxiety.

RLS is a neurological condition defined as an uncomfortable urge to move the legs, especially during bedtime, and is worsened by rest and improved by movement (10). RLS is thought to be a common, albeit underdiagnosed, disorder (11). Globally, RLS is estimated to affect about 5–15% of the population (12). A recent study in Saudi Arabia showed that RLS affects about 5.6% of the population (13, 14). The pathogenesis of RLS is still uncertain, but some evidence suggests a potential role of intracortical inhibition and a dysfunctional dopaminergic system (3, 15). The cause of RLS in most cases is unknown and considered idiopathic, while others are secondary to an underlying condition such as iron deficiency and end-stage renal disease (16, 17). Other secondary causes include neurological disorders such as diabetic peripheral neuropathy (18). RLS is a significant cause of poor sleep quality and daytime somnolence, and fatigue (19). It is diagnosed clinically by meeting the four criteria provided by the IRLSSG (20). Multiple studies pointed out the increased prevalence of RLS in patients with MS, although it is unknown if MS can be considered a direct secondary cause of RLS (21–27). Furthermore, the relationship between RLS, poor sleep, and fatigue in pwMS requires further examination (4, 28, 29).

The majority of published literature on RLS in MS comes from the Western world, with only a few studies from the middle east (30, 31). This is important because current evidence suggests that RLS is genetically based and that racial differences in RLS may exist (32). RLS frequency and predictors vary by ethnicity, according to reports (33). Ethnic disparities in the prevalence of RLS among MS patients have been reported, too (34). There is no published literature on the prevalence of RLS among pwMS in Saudi Arabia.

The primary objective of this study is to estimate the prevalence of RLS among Arab pwMS of variable ages and genders. The presence or absence of any correlation between RLS symptoms and MS will be investigated. The study will also explore whether the duration of MS and age play a role in RLS. To study the impact of RLS, the study will further clarify the relationship between RLS symptoms, sleep disturbance, and daytime fatigue in pwMS.

## 2. Materials and methods

### 2.1. Ethical consideration

The study was approved by the Institutional Review Board (IRB) at King Saud University (approval number E-20-5,448). All the participants were 20 years of age or older. All participants consented to participate in the study.

### 2.2. Population

We conducted a case–control cross-sectional study at King Saud University Medical City between September 2020 and September 2021. Patients with a diagnosis of multiple sclerosis (20 years of age or older) attending the MS clinic were contacted. We excluded pregnant women, patients with diabetes mellitus, chronic kidney disease, rheumatologic disorders, documented mental health disorders, or patients receiving antidepressants (based on individuals’ medical records) (18, 35). Participants who had previously received a sleep problem diagnosis (reported by the participant or gleaned from the participant’s medical records) were also excluded. Those conditions were excluded as they are considered risk factors for RLS or can produce RLS-like symptoms.

We further conducted our study on an age-matched control group of healthy participants. We used the same exclusion criteria to rule out different cofounders and tried isolating MS as the main variable.

A minimum of 110 participants was set to get enrolled in the study. The sample size was calculated based on previous literature that estimated the prevalence of RLS in MS to be 14.4%, so a proportion of 14.4% was taken with alpha (α) of 0.05 and a precision of 7% (24). The estimated sample size was 92 participants. An additional 20% was added to compensate for any nonresponses or excluded participants. This resulted in a total sample size of 110 per group. The sample was then randomly selected from a set of patients already diagnosed with MS. Subjects in the control group were recruited using advertising on the social communication platforms that is distributed to the public.

### 2.3. Procedures

Patients with MS were contacted by phone, and the questionnaire was administered by trained researchers. This methodology has the advantage of ensuring the patients’ full understanding of the questions and the completeness of the data collected. We explained the study to the participants and obtained informed consent. The participants were interviewed using a preset standardized questionnaire to measure the prevalence of RLS and fatigue and to assess the sleep quality of those patients. The questionnaire included the International Restless Legs Syndrome Study Group (IRLSSG) (13) diagnostic criteria for RLS, Fatigue Severity Scale (FSS) (36), and Pittsburgh Sleep Quality Index (PSQI) (37). Those questionnaires were available and validated in both Arabic and English based on the patient’s preference. To ensure the privacy of the participants, no names or IDs were recorded on the form. The control group was interviewed using an online survey. The survey started with informed consent and contact information in case any participant had any inquiries. This was followed by questions about the presence of any exclusion criteria. Further analysis was carried out with the same parameters to compare the controls with our MS sample regarding the prevalence of RLS and fatigue and to assess the sleep quality of those participants using the same questionnaires for the MS group.

To establish and calculate the severity of RLS, we used the internationally accepted IRLSSG diagnostic criteria. For the diagnosis of RLS, participants had to meet all four essential criteria, which are (20, 38):

1. The urge to move one’s legs is usually, but not always, accompanied by an uncomfortable sensation in the legs.
2. The urge to move the legs and uncomfortable sensations worsen during rest or inactivity.
3. The urge to move the legs and uncomfortable sensations are relieved by movement.
4. The urge to move the legs and uncomfortable sensations only occur or are worse during the evening or night rather than during the day.

After meeting all four criteria, participants were asked 10 questions to assess the severity of RLS. Each question was rated from very severe (4) to none (0), and the sum out of 40 was calculated. Participants were categorized based on their scores. A score of 1–10 meant that the person suffered from mild RLS, 11–20 moderate, 21–30 severe, and 31–40 very severe (39).

The Fatigue Severity Scale is a validated tool used to measure fatigue severity, which consists of 9 items. Participants were instructed to choose a number between 1 (Strongly disagree) and 7 (Strongly agree) for each item. After providing a number for each statement, the mean was calculated. Participants with a mean score of more than or equal to 4 are considered to have fatigue, while a score of less than four is considered fatigue-free. The cutoff of 4 was obtained from the literature review (40).

The PSQI is used to calculate the quality of a person’s sleep. It is composed of 10 questions, which are used to calculate seven different components. The 10 questions cover several elements of sleep, such as time to bed, time until actual sleep, amount of sleep, time out of bed, and sleep disturbances. After answering the 10 questions, each component is given a score from 0 to 3, with 0 indicating no difficulty and 3 indicating severe difficulty. Then the sum of all components is calculated, ranging from 0 to 21. A higher score indicates worse sleep quality (41).

### 2.4. Statistical analysis

Data collected were analyzed by using SPSS 28.00 (Chicago, IL, United States) and MS Excel 16.46 (Microsoft, Redmond, WA, United States) to analyze the data. Both cross-sectional and descriptive statistical tools were employed to articulate the collected data. The cross-sectional analysis included prevalence and prevalence odds ratio. Descriptive analysis included mean, standard deviation, frequency, and percentages. Student t-test, Mann–Whitney U test (if normality test failed), or Chi-square statistical tests were used as appropriate to analyze bivariate data. A *p*-value of <0.05 with a 95% CI was used as a threshold to measure the significance and precision of the results.

We performed multivariable logistic regression analysis to examine the association between MS and RLS and the effect of age and sex as independent variables. The same methodology was used to investigate potential risk factors for RLS among patients with MS, such as age, sex, and disease duration. Linear regression was used to investigate the association between RLS scores and fatigue scores using age, sex, and MS disease duration as the independent variables. A value of *p* < 0.05 is considered statistically significant.

## 3. Results

One hundred and twenty-three (123) pwMS were contacted. One hundred nine patients were eligible to be included in the study. The other 14 patients (11.38%) were excluded due to the presence of exclusion criteria, unwillingness to participate, or not responding to the call. Any missing data collected from any participant resulted in the exclusion of the participant from further analysis too. Of the excluded patients, 5 were diabetics, eight were receiving antidepressants, and one woman was pregnant. The socio-demographic analysis of the study showed that out of the 109 participants, 71 were females (65.1%), and 38 were males (34.9%). The mean age of the participants was 31.9 ± 8.7 years. The average MS disease duration of all participants was 69.4 ± 59.5 months (Table 1).

**Table 1:** Demographics and RLS prevalence comparing pwMS to controls.

Item	MS population (*N* = 109)	Control population (*N* = 216)
Male; n (%)	38 (34.9%)	105 (48.6%)
Age in years (mean ± SD)	31.9 ± 8.7 years	30.4 ± 9.9 years
MS duration (mean ± SD)	69.4 ± 59.5 months	–
Person with RLS n (%)	33 (30.3%)	18 (8.3%)
RLS categories		
Mild, n (%)	9 (27.3%)	4 (22.2%)
Moderate, n (%)	12 (36.4%)	8 (44.4%)
Severe, n (%)	11 (33.3%)	5 (27.8%)
Very severe, n (%)	1 (3%)	1 (5.6%)

MS, Multiple Sclerosis; RLS, Restless Leg Syndrome.

In the control group, 255 participants attempted to complete the questionnaire, but only 216 were eligible to be included. The other 39 participants (15.3%) were excluded due to the presence of exclusion criteria or missing data. The socio-demographic analysis of the study showed that out of the 216 participants, 111 were females (51.4%), and 105 were males (48.6%). The mean age of the participants was 30.4 ± 9.9 years.

As shown in Table 1, the prevalence of RLS in pwMS was 30.3% (*N* = 33), compared to 8.3% in the healthy control group. Compared to controls, patients with MS had five times higher odds of having RLS, even after adjusting for age and sex (OR 5.1, *p* < 0.005, 95% CI 2.7–9.8). In terms of RLS severity, nine pwMS (27.3%) had mild RLS, 12 (36.4%) had moderate RLS and 11 (33.3%) with severe disease. Among controls, the majority had moderate disease (44.4%), 4 (22.2%) had mild disease, and the remaining were categorized as severe or very severe.

During the descriptive and inferential analysis of the relation between age, MS duration, and the presence of RLS shown in Table 2, the mean MS duration for RLS-positive patients was 67.8 (± 58.4) months, while for RLS-negative patients was 70.1 (± 60.4) months. In the multivariable model, we found no statistically significant association between the MS disease duration and RLS status. Regarding the relationship between patient age and the presence of RLS, the mean age for pwMS and RLS was 35.9 (± 9.7), while it was 30.2 (± 7.6) for MS/RLS negative patients (OR 1.1, *p* = 0.001; 95% CI 1.04 and 1.2; Table 2). However, patient gender was not associated with having RLS.

**Table 2:** Comparison of mean values of age and MS duration in relation to RLS.

	MS population	*p-*value	Control population	*p*-value
Age (years)				
With RLS	35.9 ± 9.7	0.001	30.4 ± 10.5	0.99
Without RLS	30.2 ± 7.6		30.4 ± 9.9	
MS duration (months), mean ± SD				
With RLS	67.8 ± 58.4	0.85	N/A	
Without RLS	70.1 ± 60.4			

MS, Multiple Sclerosis; RLS, Restless Leg Syndrome.

The presence of fatigue was studied using the FSS module with a cutoff score of 4 to determine whether a patient has significant fatigue. The presence of fatigue was much higher (57.6%) in pwMS and RLS compared to pwMS and but not RLS (32.9%) in MS/RLS negative patients. The mean FSS score was higher in pwMS with RLS (4.1 ± 1.4) compared to pwMS without RLS (3.3 ± 1.6), and this was statistically significant (*p* = 0.008). For the controls, the mean FSS score in RLS-positive participants was 4.6 (± 1.5), while the mean FSS score in RLS-negative participants was 3.2 (± 1.4). An odds ratio of the presence of fatigue in RLS-positive patients was estimated to be 2.8 (*p* = 0.02, 95% CI of [1.2; 6.4]) (see Table 3). In the multivariable model, after accounting for age, sex, and disease duration, we observed that pwMS with RLS were at increased risk of having fatigue compared to pwMS without RLS (OR 3.6, *p* = 0.008, 95% CI of [1.4; 9.3]). In the linear model, we found that a 1-point increase in the RLS was associated with a 0.07-point increase in the FSS after adjusting for age, sex, and disease duration (*p* < 0.001, 95%CI 0.03–0.1).

**Table 3:** Analysis of the association between RLS status and fatigue in MS Sample.

	MS Population			Control population		
RLS status	With RLS (*N* = 33)	Without RLS (*N* = 76)		With RLS (*N* = 18)	Without RLS (*N* = 198)	
With fatigue (FSS ≥ 4)	19 (57.6%)	25 (32.9%)	OR = 2.8 95%, CI = 1.2–6.4, *p* = 0.02	15 (83.3%)	64 (32.3%)	OR = 10.5, 95% CI = 2.9–37.5, *p* < 0.001
Without fatigue (FSS < 4)	14 (42.4%)	51 (67.1%)		3 (16.7%)	134 (67.7%)	
FSS Score (mean + SD)	4.1 ± 1.4	3.3 ± 1.6	*p* = 0.008	4.6 ± 1.5	3.2 ± 1.4	*p* < 0.001

MS, Multiple Sclerosis; RLS, Restless Leg Syndrome; FSS, Fatigue Severity Scale.

For the analysis of sleep quality and disturbance, the Pittsburgh Sleep Quality Index was used. A bivariate analysis of the association between sleep disturbance and RLS is shown in Table 4. The average Global PSQI score for PwMS with RLS (33 participants) was 6.3 (± 4.1), and for PwMS without RLS (76 participants) was 5.6 (± 3.4). However, this was not statistically significant (*p* = 0.4; 95% CI of [−2.2;0.9]). Further analysis of sleep quality and disturbance among the controls yielded an average Global PSQI score for controls with RLS was 8.3 (± 3.7) and 5.1 (± 3.6) for controls without RLS.

**Table 4:** Comparison of sleep disturbance values in relation to RLS status.

	MS population					Control population				
RLS status	With RLS (*N* = 33)	Without RLS (*N* = 76)		*p*-value		With RLS (*N* = 18)	Without RLS (*N* = 198)		*P*-value	
Global PSQI score (mean ± SD)	6.3 ± 4.1	5.6 ± 3.4		0.4		8.3 ± 3.7	5.1 ± 3.6		<0.001	
PSQI components scores										
Sleep quality (mean ± SD)	1.1 ± 1.1	0.9 ± 0.9		0.2		1.6 ± 1.0	0.9 ± 1.0		0.007	
Sleep latency (mean ± SD)	1.6 ± 1.2	1.4 ± 1.2		0.3		1.8 ± 1.2	1.4 ± 1.1		0.17	
Sleep duration (mean ± SD)	0.9 ± 0.98	0.8 ± 0.8		0.5		1.2 ± 0.9	0.7 ± 0.9		0.023	
Sleep efficiency (mean ± SD)	0.48 ± 0.9	0.7 ± 1.1		0.3		0.8 ± 1.1	0.4 ± 0.8		0.09	
Sleep disturbance (mean ± SD)	1.21 ± 0.6	1.0 ± 0.5		0.047		1.5 ± 0.7	0.9 ± 0.6		<0.001	
Sleep medication (mean ± SD)	0.18 ± 0.6	0.21 ± 0.6		0.8		0.1 ± 0.3	0.2 ± 0.5		0.078	
Daytime dysfunction (mean ± SD)	0.79 ± 0.9	0.71 ± 0.9		0.7		1.4 ± 0.9	0.7 ± 0.8		<0.001	
Time variables										
	Mean	*SD*	Mean	*SD*	*P*-value	Mean	*SD*	Mean	*SD*	*P*-value
Bedtime (hh:mm)	8:46	9:37	6:24	8:29	0.2	4:31	5:07	5:45	7:31	0.50
Latency (mm.ss)	51.12	57.06	32.54	34.19	0.04	63.11	112.7	38.52	39.00	0.04
Get up (hh:mm)	9:15	2:15	9:39	3:09	0.5	10:44	2:34	9:56	3:00	0.28
Sleep duration (hh:mm)	7:07	1:54	7:14	1:44	0.8	6:28	1:46	7:35	2:08	0.03

RLS, Restless Leg Syndrome; PSQI, Pittsburgh Sleep Quality Index.

Different components of the PSQI score were also analyzed. It is apparent from Table 4 that for the sleep disturbance component, the mean score for pwMS and RLS was 1.2 (± 0.6) and was 1 (± 0.5) for pwMS without RLS (*p* < 0.05; 95% CI of [−0.42; −0.003]), shows that pwMS with RLS suffered more sleep disturbances. However, the difference in the results of sleep quality, latency score, sleep duration, sleep efficiency, and daytime dysfunction components were minor and did not reach statistical significance (Table 4). In line with the MS group, the healthy controls with RLS had a mean disturbance score of 1.5 (±0.7), while controls without RLS had a lower score of (0.9 ± 0.6) (*p* < 0.001; 95% CI of [−0.31; −0.93]).

## 4. Discussion

This study aimed to determine the prevalence of RLS in Arab pwMS, as well as the correlation between RLS and poor sleep quality and fatigue among PwMS. The prevalence of RLS among pwMS was 30.3%, which gives a higher likelihood of having RLS [*p* < 0.001, odds ratio (OR) = 4.8, 95% confidence interval (CI) 2.5–8.9]. This is similar to a previously published small study from Egypt (20%) and slightly higher than reported by an Iranian study (27.8%) (30, 42). It is clear from this study and the previous studies that the prevalence of RLS among pwMS is much higher than that of the general populations from different ethnic groups and geographic locations. This raises the possibility of a causative relationship between MS and RLS and calls for more research to explore the association (21, 43). Especially given that a connection between MS and RLS has justification. The dopaminergic system is assumed to be dysfunctional in current theories of RLS (44). Additionally, it has been proposed that RLS may result from dopaminergic system failure caused by demyelination of A11 neurons in the hypothalamus, which regulate spinal excitability and sensory processing of leg afferents (45, 46). This association may have a convincing explanation in MS-related spinal cord injury, which can disrupt ascending or descending pathways and cause a brain-spinal cord dissociation, resulting in RLS. Clinical and neurophysiological research has shown that spinal motor hyperexcitability in RLS may result from impairment in a descending cerebrospinal inhibitory circuit (34, 47, 48). In addition, dopaminergic descending neurons that extend from the A11 hypothalamic area to D3 receptors found in the dorsal and intermediolateralis spinal nuclei may also be impacted by a spinal lesion associated with MS (49). In either scenario, dopaminergic dysfunction brought on by MS-related spinal cord demyelination may cause RLS. Another factor that could be involved in RLS symptomatology is iron. Low iron stores is a well-described finding in the brains of patients suffering from RLS (50). In MS, excess amounts of iron and transferrin are found in the cerebral tissues, and low iron levels were reported in the white matter of patients with MS (51). Also, low levels of blood iron have been reported in patients with multiple sclerosis (52). Therefore, it is plausible that iron dysregulation is a contributor to the symptoms of RLS and fatigue in patients with MS. The design of our study did not allow for blood extraction from cases or controls which limited the measurement of iron or ferritin levels. These factors emphasize the need for additional investigation, particularly in the areas of pathology and radiology studies, to clarify the potential relationship between MS and RLS.

In terms of RLS severity, compared to one study conducted in Brazil, the severity of RLS in pwMS was more spread out, with about 1/3 of patients fitting each severity category (mild, moderate, or severe) (25).

We did not find a significant relationship between the impact of MS duration on the development of RLS. This is consistent with previous research, which could not find any association between RLS and MS duration (26). However, age did seem to impact the development of RLS as pwMS with RLS tends to be older. This is consistent with studies in RLS that revealed that RLS is a disorder that increases in prevalence with increasing age, although it can occur at any age (12). Fatigue prevalence was significantly higher among pwMS who suffer from RLS (57.6%) compared to pwMS without RLS (32.9%). This is of interest as it could indicate that RLS could be significantly contributing to fatigue symptoms among patients with MS. However, care should be considered in the interpretation of these results, as the association between RLS and fatigue is more complex because other unknown or unstudied factors can be contributing to both fatigue and RLS in the same patient.

This study affirms the finding that sleep quality (measured by PSQI) is impaired among pwMS, especially those who suffer from RLS symptoms. When looking at the different components of the PSQI, RLS patients had higher sleep latency score (1.6 (±1.2), mean duration of 51.12 min) and sleep disturbance score of 1.2 (± 0.6) when compared to non-RLS patients (1.4 ± 1.2, mean duration of 32.5 min), 1 (± 0.5). These results show that RLS patients take a longer time to sleep and suffer more sleep disturbances when compared to non-RLS patients, and the results of our controls and past research coincide with our study (4, 5). One such research has reported that impaired sleep quality was found in 52% of its subjects (4). For the other components of the PSQI score, nothing was significantly different between the two groups.

There were some limitations to this study. First, the data was collected during the COVID-19 pandemic, which required the use of the phone to gather information. This resulted in a higher number of non-respondents or patients refusing to participate. Second, the nature of the study did not allow exploration of other factors that influence RLS symptoms, such as disability scores, MS disease severity, etc. Third, while the sample size was adequate to determine the general prevalence of RLS in pwMS, the number of patients with RLS was too small to better assess associations. However, this did not seem to significantly affect the results, as the majority fell in line with previously published results. In this study, the impact of RLS on the quality of life was not examined. Poor sleep has been previously shown to be an independent predictor for worse quality of life among patients with MS (53).

In summary, this study further strengthens the association between RLS and MS. More importantly, it sheds light on the impact of RLS on sleep quality and the association between RLS and fatigue.

## 5. Conclusion

In conclusion, the prevalence of RLS among Arab patients with MS was significantly higher compared to the general population. There is a strong association between RLS and fatigue and sleep disturbances. Therefore, physicians should explore the symptoms of sleep dysfunction and RLS in MS patients, especially those who suffer fatigue, daytime somnolence, or poor sleep quality. We also recommend inquiring about RLS risk factors, such as iron deficiency and antidepressant use among those pwMS. Further research is needed to determine the impact of RLS screening and management on the quality of life and fatigue in pwMS.

## Data availability statement

The raw data supporting the conclusions of this article will be made available by the authors, without undue reservation.

## Ethics statement

The studies involving human participants were reviewed and approved by the Institutional Review Board Sub-Committee Health Sciences Colleges Research on Human Subjects, College of Medicine, King Saud University. The patients/participants provided their informed consent to participate in this study.

## Author contributions

AB, SA, and NA designed the study and reviewed and edited the final manuscript. OAld, MA, FA, BA, AA, and OAls collected the data. SA, MA, and AA analyzed the data, and it was reviewed by AB and NA. All authors read and approved the final manuscript and participated in writing—original draft preparation.

## Funding

This study was supported by the College of Medicine Research Center, Deanship of Scientific Research, King Saud University, Riyadh, Saudi Arabia.

## Conflict of interest

The authors declare that the research was conducted in the absence of any commercial or financial relationships that could be construed as a potential conflict of interest.

## Publisher’s note

All claims expressed in this article are solely those of the authors and do not necessarily represent those of their affiliated organizations, or those of the publisher, the editors and the reviewers. Any product that may be evaluated in this article, or claim that may be made by its manufacturer, is not guaranteed or endorsed by the publisher.
